# Machine learning-assisted event classification in cadmium zinc telluride positron emission tomography detectors leveraging entanglement-informed angular correlations

**DOI:** 10.1038/s41598-025-32951-6

**Published:** 2025-12-20

**Authors:** Praveen Gurunath Bharathi, Gregory Romanchek, Greyson Shoop, Michael King, Matthew Kupinski, Lars Furenlid, Shiva Abbaszadeh

**Affiliations:** 1 Department of Electrical and Computer Engineering, Baskin Engineering, UC Santa Cruz, Santa Cruz, CA USA; 2 Department of Nuclear, Plasma, and Radiological Engineering, Grainger College of Engineering, University of Illinois at Urbana-Champaign, Urbana, IL USA; 3 Department of Radiology, UMass Chan Medical School, Worcester, MA USA; 4 Wyant College of Optical Sciences, The University of Arizona, Tucson, AZ USA

**Keywords:** CZT, Machine learning, DCSc, Event classification, Mathematics and computing, Physics

## Abstract

Gamma–positron imaging with tracers that emit a prompt $\gamma$ (> 511 keV) is vulnerable to Compton down-scatter leaking into the 511-keV window and mimicking true annihilation pairs. Conventional Positron Emission Tomography (PET) systems reconstruct annihilation events without leveraging that the two 511-keV photons are not only orthogonally polarized but also produced in a Bell-entangled state. The polarization correlations of this entanglement imprint themselves in Compton scattering kinematics, particularly the relative azimuthal scattering angle ($\Delta \phi$), offering a physics-informed handle for event discrimination. We present a machine-learning framework that exploits these quantum-encoded features to resolve true lines of response (LORs) and reject random coincidences in a dual-panel cadmium zinc telluride (CZT) system. Detected events were categorized into one-photoelectric (1P) and Compton (1C) interaction patterns, yielding four candidate interaction sequences per event. Each event was represented as a 4 $\times$ 21 feature matrix comprising spatial coordinates, energy deposits, and angular descriptors, including $\Delta \phi$ and polar scattering angle $\theta$. Feature ablation with five-fold cross-validation revealed that the combination of energy and $\Delta \phi$ provided the highest discriminative power (Area Under the Receiver Operating Characteristic Curve (ROC–AUC) 0.87–0.95), followed by energy alone (ROC–AUC 0.85–0.95), while inclusion of spatial coordinates with energy and $\Delta \phi$ ranked third, achieving consistent performance across folds (ROC–AUC 0.81–0.91). These results demonstrate that incorporating entanglement-sensitive angular features into learning pipelines can suppress prompt contamination while preserving true LORs in a gamma-positron imaging system.

## Introduction

Positron Emission Tomography (PET) is an important diagnostic imaging tool in nuclear medicine that allows in vivo imaging of cancerous cellular tissue which is commonly imaged using radiopharmaceuticals containing the radionuclide $^{18}$F. PET achieves this through the use of opposite-facing gamma ($\gamma$)-ray detectors that detect the absorption of back-to-back pairs of 511 keV annihilation photons that originate from positrons $(\beta ^+)$ emitted as a consequence of the target radionuclide’s radioactive decay process. The imaginary line connecting these two detection points is called the line of response (LOR), which is used to reconstruct the source position during image formation. Improvements in nuclear medicine have seen the use of radionuclides beyond $^{18}$F for applications in targeted radionuclide therapy (TRT) in cancer treatment which involve various non-pure $\beta ^+$ emitters that contain high-energy $\gamma$-rays capable of degrading qualitative and quantitative imaging performance^1^.

In contrast to $^{18}$F, which primarily emits $\beta ^+$, commonly used non-pure $\beta ^+$ emitters such as $^{68}$Ga and $^{44}$Sc provide emissions of high energy $\gamma$’s (> 511 keV) that can scatter and lose enough energy to be detected with similar energy as annihilation photons which degrades the imaging performance^2,3^. These additional $\gamma$’s, often called prompt $\gamma$’s, are emitted nearly simultaneously with $\beta ^+$ during the decay process. Prompt $\gamma$’s can lead to a potential increase in system dead time as well as an increase in uncorrelated coincidences that make activity concentration measurements of non-pure $\beta ^+$ emitting isotopes more problematic than with $^{18}$F^4–6^. One final consideration involves the range at which a $\beta ^+$ may travel from non-pure emitters. Imaging higher energy $\beta ^+$ emitters can demonstrate less sharp features due to the larger ranges at which the $\beta ^+$ travels away from the true site of the radio distribution before annihilation limiting high resolution PET and quantitative accuracy in small regions^7–9^.

Multiple innovations in PET over the past decade have emerged to take advantage of the prompt $\gamma$’s from non-pure $\beta ^+$ isotopes to push the limits of PET. Solutions in data processing have been demonstrated in the implementation of PET imaging of multiple isotopes by way of imaging a pure $\beta ^+$ emitting isotope with a non-pure prompt $\gamma$ emitting isotope^10,11^. In multi-isotope PET with prompt-$\gamma$ detection, all events are acquired simultaneously and separated offline using coincidence time windows. The list-mode data are sorted into two datasets for special reconstruction algorithms: one containing only standard annihilation photon coincidences and another containing triple coincidences of annihilation photons with the prompt $\gamma$^12,13^.

Where multiple isotope imaging can be implemented in conventional PET systems, further improvements to PET system technology include taking full advantage of the information that prompt $\gamma$’s contain through their scattering kinematics^14–16^. When considering PET, for high Z materials like Cadmium Zinc Telluride (CZT), a higher fraction 511 keV photons scatter and are subsequently detected as opposed to full energy absorption through photoelectric absorption which can lead to an increase in system sensitivity by reconstructing the LOR from Compton scattered annihilation photons^17^. Additionally, combining the principles of Compton Camera imaging of prompt $\gamma$’s with the coincident detection of annihilation photons leverages what was once unfavorable $\gamma$ radiation of non-pure emitters into useful information to correct for $\beta ^+$ range effects. This is possible through the recovery of the sequence of events in which a prompt gamma scatters within the detector and then deposits its remaining energy in the form of photoelectric absorption. Through simultaneous detection of annihilation photon coincidences and prompt $\gamma$ Compton scattered event information we can increase system sensitivity, however any image resolution improvements of non-pure $\beta ^{+}$ emitters would be dependent on detector physics limitations of the Compton Camera scattering material of the system such as doppler broadening and energy resolution^14,18,19^.

The additional utility of prompt $\gamma$’s has been leveraged in the implementation of positronium lifetime imaging^20,21^. When a $\beta ^+$ is emitted, it has the opportunity to form one of two different forms of positronium, para-positronium (p-Ps) or ortho-positronium (o-Ps). The average lifetime, the time between $\beta ^+$ decay and annihilation, of p-Ps and o-Ps has been demonstrated to depend on the surrounding tissue environment which opens the door for additional biomarking of cellular tissue^22,23^.

**Fig. 1:**
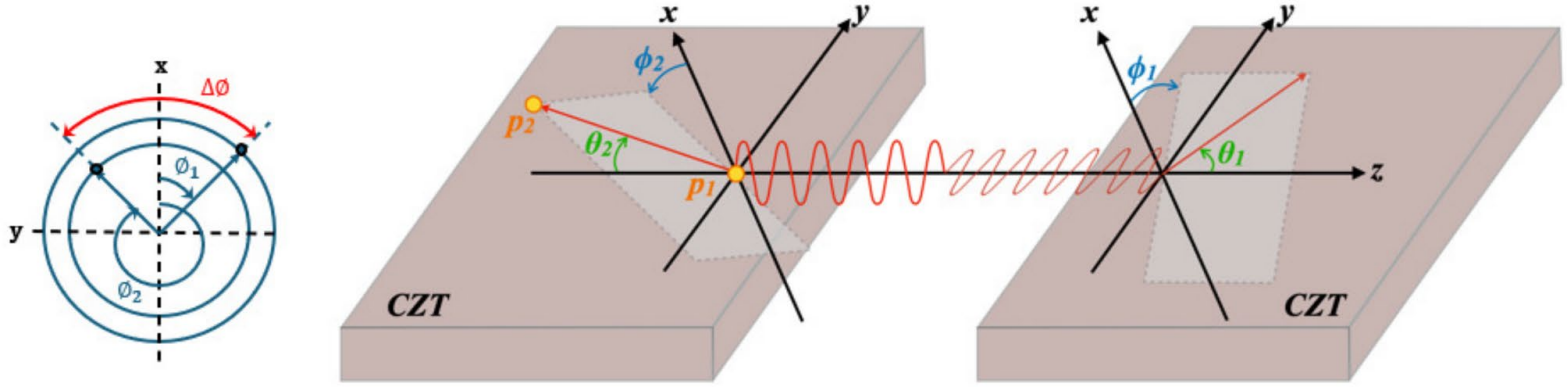
DCSc event; In addition to the polar scattering angles of the two scattered annihilation photons ($\theta _1, \theta _2$), the planes on which both annihilation photons scatter can be computed from their azimuthal angles ($\phi _1,\phi _2$).

What the various methods of detecting prompt $\gamma$’s of non-pure emitters provide is means to increase the sensitivity of conventional PET systems. This need to increase system sensitivity cannot be stressed more strongly with the emergence of imaging that utilizes the quantum entanglement (QE) properties of annihilation photons with the aim of performing QE-enhanced low-noise imaging^24^. This requires the detection of coincident annihilation photons that both undergo Compton scattering followed by photoelectric absorption in what is defined as a Double Compton Scatter coincidence (DCSc) event as visually depicted in Fig. 1. In a coordinate space where the LOR of the annihilation photons defines the z-axis, when the polar angles ($\theta _1,\theta _2$) of both photons from a DCSc scatter near 81.7$^\circ$, the distribution of the detected DCSc will demonstrate a maximum probability of having orthogonally oriented scattering planes ($\Delta \phi = \pm 90$) determined by their relative difference of their azimuthal scattering angles ($\phi _1,\phi _2$)^25–30^.

The success of imaging systems to recover the scattering information of high energy $\gamma$’s lies in the ability of a system to determine the correct sequence of scattering, that is the first Compton scattering event location and the subsequent photoelectric absorption event. Typically, systems have been constructed as dual-layer systems where a conventional cylindrical PET detector serves as the absorption layer with a scattering layer inserted between the absorption layer and imaging field of view (FOV)^31–33^. However, semiconductor detectors such as CZT are high Z materials that can provide a high cross-section of Compton scattering interactions with $\gamma$’s and, in the case of edge-on CZT, provide enough thickness for the subsequent detection of intra-scattered $\gamma$’s^17^. A major drawback of the single-layer approach is the ambiguity introduced by multiple-interaction photon events (MIPEs), which makes accurate recovery of the true interaction sequence essential. In the realm of Compton cameras, simple energy discrimination methods derived from Monte Carlo simulations show that for incident $\gamma$’s between 400 and 662 keV have a success rate ranging from 50 to 70% for correctly identifying the interaction sequence^34^.

**Fig. 2:**
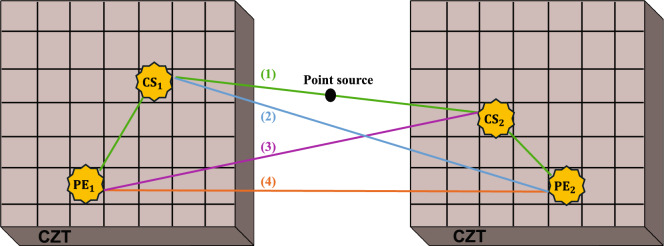
Visualization of four potential DCSc between two detectors. Line (1) in green represents the true coincidence by pairing the correct Compton scattering event (CS$_1$) with photoelectric event (PE$_1$) in one detector and pairing the correct Compton scattering event (CS$_2$) with photoelectric event (PE$_2$) in the other. Coincidences (2), (3), and (4) in the blue, purple, and orange are potential incorrectly determined coincidences..

Considering the scenario of a DCSc coincidence event as depicted in Fig. 2, we can see that this ambiguity in the recovery of the potential sequences of interactions for MIPEs will lead to qualitatively large differences in LOR estimation which can further degrade the performance of a system to accurately implement QE information. All of these considerations assume that the DCSc event is true, which will not always be true, especially when considering the imaging of non-pure emitters. However, given that there is a correlation in $\Delta \phi$ for DCSc events, this information could be used to differentiate false coincidences of annihilation photons with prompt $\gamma$’s. This increase in information gathered from $\gamma$’s could possibly be utilized in machine learning (ML) algorithms to properly identify the classification of DCSc events from prompt $\gamma$ coincidences.

ML algorithms are increasingly applied to PET detectors to improve spatial resolution and correct for inter-crystal scattering (ICS), which often limits image quality in high-resolution systems. Deep neural networks (DNNs) have been used for more precise event positioning and for identifying ICS in multiplexed PET detectors^35^. Similarly, convolutional neural networks (CNNs) have been implemented for ICS recovery, with some approaches using networks like ICS-Net^36^ or a 1D U-Net^37^ to predict the first interaction position of a photon, thereby improving positioning accuracy. In other applications, ML positioning algorithms such as Extreme Gradient Boosting (XGBoost) are used to enhance spatial resolution in semi-monolithic scintillator detectors, with hyperparameter optimization performed using genetic algorithms or particle swarm optimization^38^.

Despite these advances, ICS remains a major challenge for accurate coincidence event classification, particularly in high-resolution systems where DCSc events can be misclassified as true coincidences. Such misclassifications can degrade image quality by introducing false prompts. To address this limitation, we propose a ML–based approach for event classification by explicitly rejecting false coincidences at the event level.

## Materials and methods

### Dataset overview

We generated the underlying dataset used for training, validation, and testing of the ML model from a pair of Monte Carlo physics simulations via GATE. GATE is a well-used medical imaging simulator based on the GEANT4 toolkit^39,40^. We performed the simulations in GATE v9.3 as the GEANT4 v11.1.1 dependency includes a physics module for entanglement-born scattering kinematics of annihilation $\gamma$-rays^26^. These simulations, the raw and processed data, and processing steps are the same as those used in Romanchek et al.^41^. We follow with a description of the simulation parameters, post-processing steps, and final dataset construction.

### GATE simulation overview

We performed two GATE simulations to model a simplified Sc-44 source, a dual emitter of prompt $\gamma$-rays (1157 keV with 99.875% emission rate) and $\beta ^+$’s (98.97% emission rate). Rather than model the complex decay mechanics of the Sc-44 isotope, our simplified source always emits the 1157 keV prompt $\gamma$’s and $\beta ^+$’s (100% emission rate for both particles). Further, we simulated each particle type separately and then paired the independent decay data together using a priori information (e.g., events associated with decay index N from the prompt $\gamma$-ray simulation were grouped with events in the $\beta ^+$ simulation with the same decay index N). As the Sc-44 $\beta -\gamma$ cascade does not exhibit angular correlations, the prompt-$\gamma$ and $\beta$ emission angles can be treated as statistically independent^42,43^. It is therefore valid to simulate the prompt-$\gamma$ and $\beta$ decays separately and combine their outputs without biasing the resulting interaction distributions.

In each particle simulation, the 0.1 mm radius point source is centered in the FOV with an activity of 30 $\upmu$ Ci and half-life of 14,290 s. We conducted a 60 s simulated scan of each particle emitter and then combined and processed them with in-house code, described below.

### System description

**Fig. 3:**
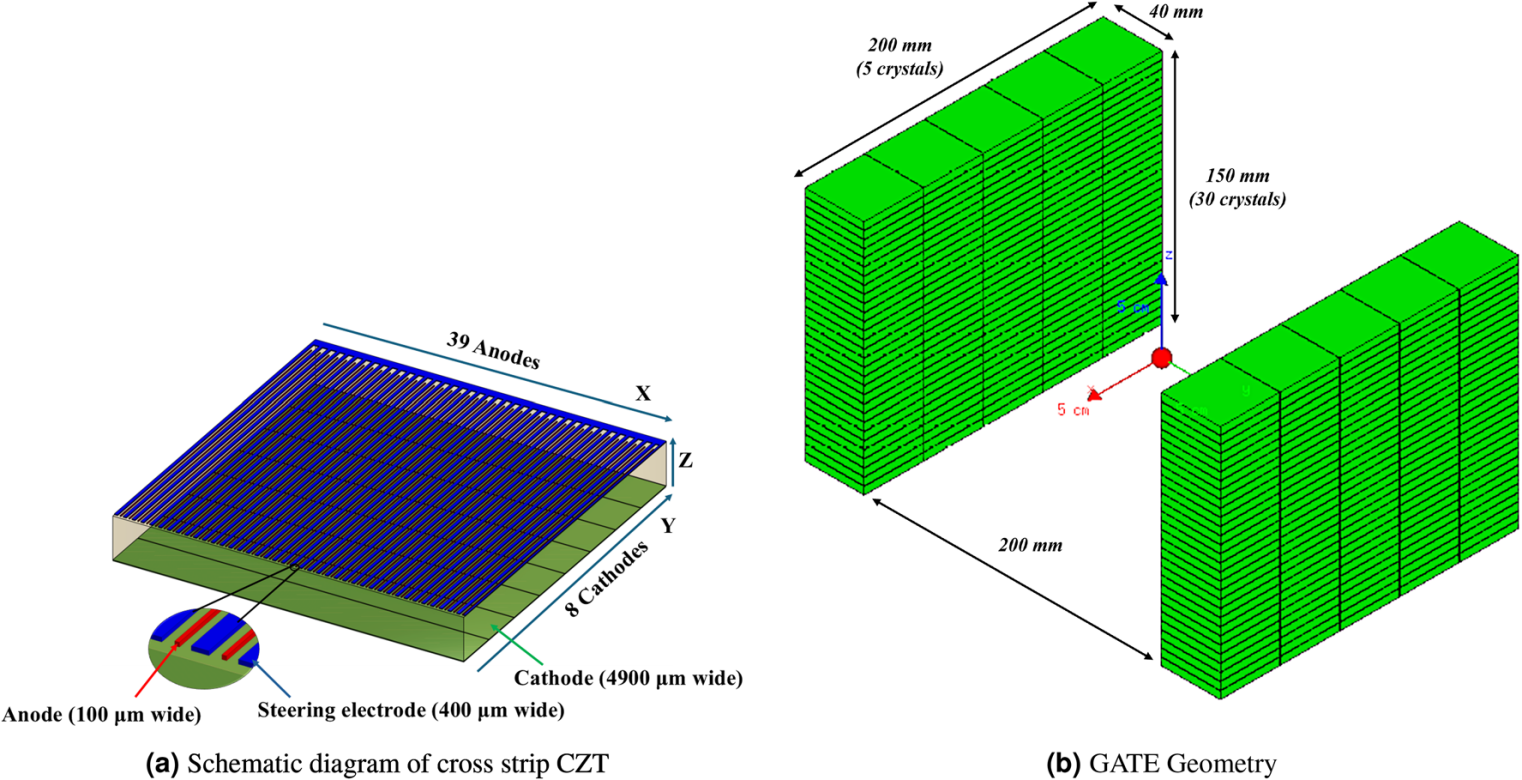
Visualization of the GATE geometry used in the simulation. The source is shown with a larger radius than used in the simulation purely for visualization.

We modeled a scaled-up version of the UCSC 2-Panel PET Scanner (2P), illustrated in Fig. 3 in GATE geometry. The system geometry consists of two opposing 15$\times$20 cm² detector panels, each containing a 5$\times$30 array of edge-on oriented 150 CZT crystals ($40\times 5\times 40 mm^3$).

While the experimental system uses a cross-strip architecture for its electrodes, the simulated system approximates this setup with a pixelated detector assumption. With cathodes running along the x-axis on one side of the CZT crystal, and anodes running along the y-axis on the other side, the cross-strip architecture uses activated electrode pairs to yield x-y event positions. Then, the ratio of the electrode energies is used to acquire the depth (z) of each interaction. In short, the near millimeter resolution event positioning provided from the cross-strip architecture is modeled as 1 mm pixels spanning the x-, y-, and z-dimensions of each crystal. This bypasses the need to model charge transport within the semiconductor crystals while still providing a reasonable statistical model of the system. Greater system details are available^44–47^.

### Event processing and dataset definition

The GATE simulations generate *Hit* files containing time-ordered, ground-truth records for every particle-detector interaction in the simulation. The data processing workflow consisted of five stages:

1. Dataset Combination. Prompt $\gamma$-ray emissions from the prompt-$\gamma$ simulation were paired with $\beta ^+$ emissions from the annihilation simulation by matching their shared eventID (decay index).
2. Event Grouping. For each decay, all associated photon interactions were collected. Each decay may produce up to three photons (one prompt $\gamma$ and two annihilation $\gamma$’s), and each photon may undergo MIPE.
3. Event Filtering. For each event group, we keep only those for which all three photons are detected and where each photon undergoes a MIPE, i.e., those capable of forming a DCSc regardless of which two photons are paired.
4. Blurring and Binning. Energies were then blurred with a Gaussian kernel to match the system energy resolution (5.85% at 511 keV ^45^), and interaction positions were discretized to 1 mm pixel centers in *x*, *y*, and *z* to match the effective voxel size. Then, each event in each group was required to fall within a 50 keV window around 511 keV.
5. Dataset Construction. Half of the resulting samples were paired truthfully with the two annihilation photon MIPEs forming a true DCSc, and with the corresponding prompt-$\gamma$ MIPE being discarded. For the remaining samples, the prompt-$\gamma$ MIPE was paired with the annihilation photon MIPE in the opposite panel to construct a random DCSc.

This procedure yielded a total of 720,571 labeled samples, with an approximately even split between True and Random DCSc. Each sample was represented as a $4\times 21$ matrix, where each row corresponds to one candidate configuration and each column to an observable feature. The 21 features encompass the full set of measurable quantities for a given DCSc and are grouped as follows:

- Spatial coordinates (12 features): Interaction positions $(x_1,y_1,z_1)$ and $(x_3,y_3,z_3)$ from Compton scatter events, and $(x_2,y_2,z_2)$ and $(x_4,y_4,z_4)$ from photoelectric absorption events.
- Energies (4 features): Interaction energies $(E_1, E_3)$ from Compton scattering and $(E_2, E_4)$ from photoelectric absorption.
- Derived angular descriptors (5 features): Position-based polar scattering angles $(\theta _{1\textrm{pos}}, \theta _{2\textrm{pos}})$, energy-based polar scattering angles $(\theta _{1\textrm{energy}}, \theta _{2\textrm{energy}})$, and the azimuthal scattering angle difference $\Delta \phi$.

The angular features are derived quantities. While a sufficiently deep network could, in principle, infer them from the raw spatial and energy measurements, providing them explicitly ensures their information is being used.

From the interaction positions, scattering vectors were constructed and used to compute the position-derived polar scattering angles,

1 $$\begin{aligned} \theta _p = \cos ^{-1} \left( \frac{a \cdot b}{|a|\,|b|} \right) , \end{aligned}$$

where *a* is the LOR vector and *b* is the photon scattering vector. Given the interaction energies and the assumed ordering of events within each configuration, the energy-derived polar scattering angle was computed using the Compton scattering formula:

2 $$\begin{aligned} \theta _E = \cos ^{-1} \left( 1 - m_e c^2 \left( \frac{1}{E_s} - \frac{1}{E_i} \right) \right) , \end{aligned}$$

where $E_s$ is the scattered-photon energy, $E_i$ is the incident-photon energy (taken as 511 keV for annihilation photons), and $m_e c^2 = 511$ keV is the electron rest mass energy. Equation 2 does not hold for prompt $\gamma$-rays above 511 keV (e.g., 1157 keV), and this mismatch between $\theta _p$ and $\theta _E$ serves as a useful indicator of random DCSc. For cases in which $\theta _p$ becomes undefined (scatter energy $>340.7$ keV), a placeholder value of 9,999 was assigned to allow the network to easily identify and learn from these unphysical configurations.

The feature encoding entanglement information is the azimuthal angle difference, $\Delta \phi = \phi _1 - \phi _2$. While the polar angle $\theta _p$ follows directly from Eq. 1, computing $\Delta \phi$ requires evaluating the relative azimuthal orientation of the two scattering vectors with respect to the LOR. Each scattering vector was projected onto the plane orthogonal to the LOR, and the angle between these projected vectors was then extracted to determine $\Delta \phi$.

### Machine learning methods

**Fig. 4:**
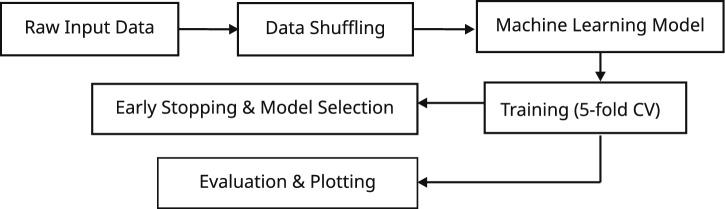
Methodology flowchart.

The overall ML methodology flowchart is shown in Fig. 4. The sample dataset consists of multiple candidate configurations for each DCSc, with each configuration represented by a fixed-length vector of features. Each candidate configuration is represented by a 21-dimensional feature vector. In total, 21 features were extracted per configuration, forming a structured input space for ML. Each event sample was thus represented by a four-row feature matrix, corresponding to the four candidate configurations. For a false LOR, none of the configurations represent a valid solution, resulting in a sample where all configurations are false. This structure allows the learning task to be naturally framed as a multi-class classification problem. To prevent positional bias during training, the order of the four rows was randomized on a per-sample basis. Furthermore, the entire dataset was globally shuffled prior to partitioning into training and validation folds. This strategy ensured statistical independence between samples and reduced the risk of overfitting to spurious ordering patterns.

A ML architecture was designed to jointly analyze the four candidate configurations associated with each event. The model input was structured as a tensor of dimension (4 $\times$ 21), where each row represents one configuration. The network employed a shared encoder module consisting of fully connected layers with nonlinear activation functions to transform each configuration into a latent representation. By applying identical encoder weights across all four inputs, the model preserved permutation invariance, ensuring that classification was independent of input ordering. The latent vectors from the four encoders were aggregated using concatenation followed by higher-order fully connected layers. This fusion stage enabled the model to evaluate competing hypotheses simultaneously, learning discriminative features that distinguished true coincidences from random ones. The final classification head output a five-dimensional probability vector, corresponding to the four true configuration classes (0–3) and a false LOR, which represented cases where none of the candidate configurations matched a valid LOR. A softmax activation was applied to normalize outputs, ensuring that the sum of probabilities equaled one. This formulation allowed the model to jointly account for both correct LOR identification and rejection of spurious candidates.

To investigate which features play the most critical role in event classification, we designed a systematic feature ablation study. Instead of relying on a single trained model, we trained 15 separate models using the same network architecture and hyperparameters, but with different subsets of the input features. For example, one model was trained using only the $\Delta \phi$ feature, another using only $\theta$, a third using the spatial coordinates, and another using only energy. We also evaluated models trained on combinations of features (e.g., $\Delta \phi$ + $\theta$, $\Delta \phi$ + spatial coordinates, $\Delta \phi$ + energy, etc.), progressively covering all meaningful subsets of the input space.

By comparing performance across these 15 models, we were able to assess the relative discriminative power of individual features and their combinations. This design allowed us to identify not only which single features were most predictive, but also how different features interacted in contributing to accurate event classification. Training was conducted using five-fold cross-validation to ensure that each fold contained a balanced representation of both true and false LORs. In this procedure, the dataset was divided into five equally sized subsets, with four used for training and one reserved for validation in each iteration. This approach provided robust performance estimates and mitigated sampling bias. Feature scaling was applied to continuous variables to normalize distributions and facilitate convergence.

**Fig. 5:**
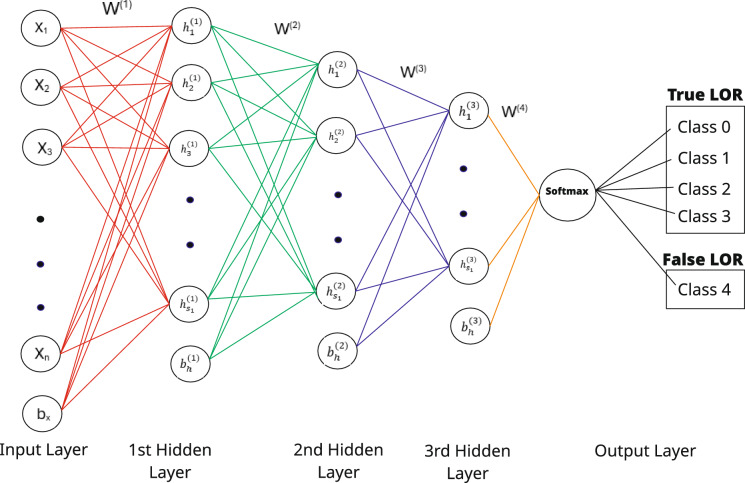
Machine learning framework.

A feed-forward neural network with three hidden layers (Fig. 5), consisting of fully connected (FC) layers with Rectified Linear Unit (ReLU) activations, was trained using the Adam optimizer^48^. The hidden layer activations $h_1^{(1)}$, $h_2^{(1)}$, ...., $h_s^{(1)}$ are the first layer features, whereas $h_1^{(2)}$, $h_2^{(2)}$, ...., $h_s^{(2)}$ are the second layer features and the final layer features are denoted by $h_1^{(3)}$, ...., $h_s^{(3)}$. ‘W’ represents the weights, ‘b’ represents the bias of the network, and ‘h’ represents the hidden layers. Focal loss with class weights was used as the objective function which addressed the class imbalance issue. Learning rate scheduling was employed to adaptively reduce the learning rate once the validation loss plateaued, thereby improving fine-tuning in later epochs. Early stopping criteria were applied based on validation loss, terminating training when no improvement was observed over a fixed number of epochs. To enhance reproducibility, model checkpoints were saved at each fold, and random seed initialization was used to ensure consistency across experiments. The overall training workflow was implemented in PyTorch^49^, leveraging graphics processing unit (GPU) acceleration for computational efficiency. The hyper-parameters used to train the model are described in Table 1.

**Table 1:** Machine learning hyper parameters.

Hyper parameter	Value/setting
Neurons in hidden layer 1	512
Neurons in hidden layer 2	256
Neurons in hidden layer 3	128
Batch size	128
Initial learning rate	$1*10^{-3}$
Optimizer	Adam
Loss function	Focal loss
K-Folds (cross-val)	5
Scheduler	ReduceLROnPlateau
Early stopping	10 epochs

Training was performed on a workstation equipped with a 3.40 GHz CPU (16 cores), 32 GB RAM, and an NVIDIA GeForce RTX 4080 GPU with 16 GB VRAM, using the PyTorch framework. Model performance was assessed using the F1-score and the area under the receiver operating characteristic curve (ROC–AUC), both derived from the counts of true positives (TP), true negatives (TN), false positives (FP), and false negatives (FN). ROC–AUC was used as the primary measure of discriminative ability, while the F1-score quantified the balance between sensitivity and precision.

ROC curves were generated for each fold and then averaged to obtain a mean ROC representation, allowing visualization of fold-specific variability and aggregate behavior. Per-fold metrics were tabulated with mean values and standard deviations. Performance was reported at two levels: (1) the row level, representing classification of individual candidate configurations, and (2) the sample level, representing event-wise classification.

## Results

**Fig. 6:**
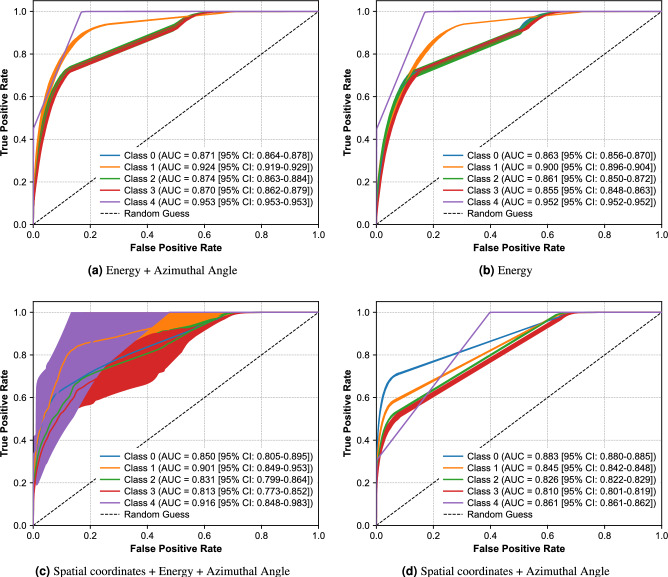
Top four best performing ROC AUC with 95% confidence intervals for feature ablations.

The ROC–AUC values (Table 2) indicate that the combination of energy and azimuthal angle difference (E+$\Delta \phi$) achieved the highest performance, with ROC AUC values ranging from 0.87 to 0.95 across classes. The next best-performing feature set was energy alone, with ROC AUC values between 0.85 and 0.95. Spatial coordinates combined with energy and $\Delta \phi$ reached values between 0.81 and 0.91, while spatial coordinates combined with $\Delta \phi$ achieved slightly lower but comparable performance. Representative ROC curves for the four highest-performing feature sets: E+$\Delta \phi$, E, *xyz*+E+$\Delta \phi$, and *xyz*+$\Delta \phi$ are shown in Fig. 6.

**Table 2:** ROC-AUC comparison for various feature combinations. Values are reported as mean ± standard deviation. A value of 1.0 represents perfect performance. The table is divided into sections for readability.

Class	Config	$\Delta \phi$	$\theta$	$\theta + \Delta \phi$	xyz	$xyz+\theta$	$xyz+\theta +\Delta \phi$	E	xyz+E
0	0	0.68 ±0.008	0.78 ±0.008	0.79 ±0.004	0.88 ±0.006	0.77 ±0.014	0.79 ±0.03	0.86 ±0.006	0.87 ±0.05
	1	0.81 ±0.006	0.72 ±0.007	0.73 ±0.005	0.84 ±0.006	0.75 ±0.014	0.77 ±0.04	0.90 ±0.003	0.88 ±0.05
	2	0.71 ±0.002	0.77 ±0.007	0.79 ±0.005	0.82 ±0.009	0.77 ±0.014	0.78 ±0.02	0.86 ±0.009	0.79 ±0.03
	3	0.67 ±0.018	0.74 ±0.01	0.76 ±0.004	0.81 ±0.01	0.74 ±0.017	0.76 ±0.04	0.85 ±0.006	0.77 ±0.03
1	4	0.91 ±0.001	0.80 ±0.0007	0.81 ±0.0005	0.86 ±0.0004	0.78 ±0.0006	0.80 ±0.05	0.95 ±0.0001	0.92 ±0.08

Class	Config	$xyz+\Delta \phi$	$xyz+E+\Delta \phi$	$xyz+E+\theta$	$xyz+E+\theta +\Delta \phi$	$E+\theta$	$E+\Delta \phi$	$E+\theta +\Delta \phi$	
0	0	0.88 ±0.001	0.85 ±0.04	0.76 ±0.03	0.82 ±0.04	0.78 ±0.02	0.87 ±0.005	0.77 ±0.01	
	1	0.84 ±0.002	0.90 ±0.04	0.76 ±0.03	0.85 ±0.06	0.77 ±0.03	0.92 ±0.004	0.75 ±0.01	
	2	0.82 ±0.002	0.83 ±0.03	0.72 ±0.06	0.82 ±0.04	0.75 ±0.03	0.87 ±0.008	0.77 ±0.01	
	3	0.81 ±0.005	0.81 ±0.03	0.74 ±0.02	0.82 ±0.05	0.73 ±0.05	0.87 ±0.006	0.75 ±0.01	
1	4	0.86 ±0.0006	0.91 ±0.05	0.79 ±0.06	0.91 ±0.08	0.81 ±0.05	0.95 ±0.0002	0.79 ±0.0006	

**Fig. 7:**
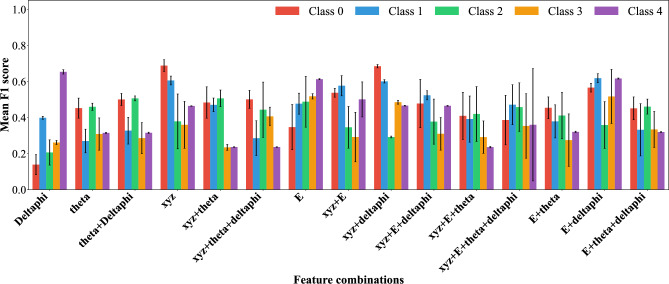
Class-wise mean F1-scores across all folds with 95% confidence intervals.

As shown in Fig. 7, the comparative evaluation of mean F1-scores across feature combinations demonstrates that energy-driven descriptors dominate classification performance. The highest overall F1-score was achieved with energy and angular separation (E+$\Delta \phi$), underscoring the strong synergy between energy deposition and angular correlation. The second-best configuration was spatial coordinates combined with angular separation (*xyz*+$\Delta \phi$), highlighting the discriminative power of geometric context when paired with angular correlations. Spatial coordinates alone (*xyz*) ranked third, while energy alone (E) provided the fourth-best performance, establishing it as the most informative single feature.

By contrast, other feature sets such as ($\theta$+$\Delta \phi$) yielded comparatively weaker performance, reflecting their limited discriminative capacity in isolation or without angular correlations. Collectively, these findings highlight that energy serves as the dominant predictor, while its integration with angular separation or spatial coordinates provides the most robust and balanced framework for $\gamma$ event classification.

**Fig. 8:**
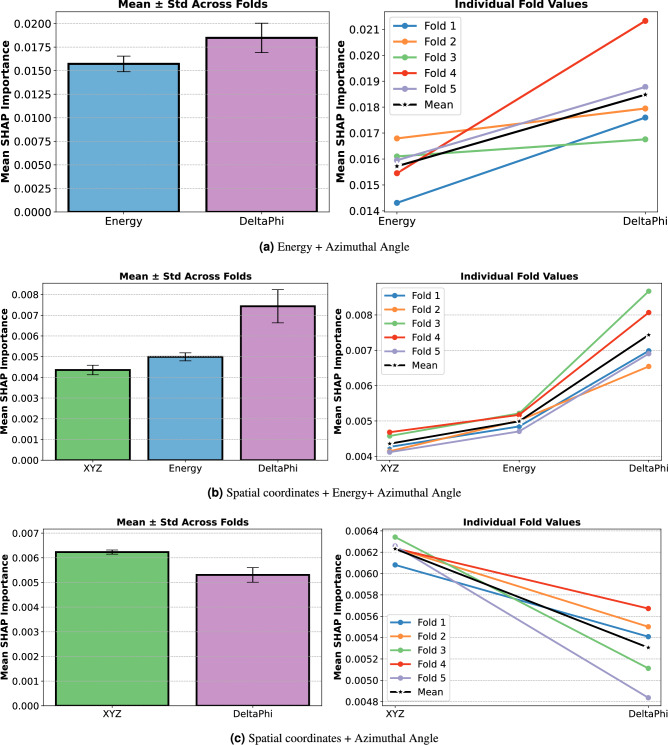
SHAP feature importance for the top-performing feature combinations. Panels show mean ± standard deviation across folds (left) and fold-wise values (right) for: (**a**) energy + $\Delta \phi$; (**b**) spatial coordinates + energy + $\Delta \phi$; and (**c**) spatial coordinates + $\Delta \phi$.

To quantify the relative contributions of individual features within the highest-performing configurations identified in Fig. 6, SHapley Additive exPlanations (SHAP)^50^ values were computed for each fold of the five-fold cross-validation. The resulting feature importances are summarized in Fig. 8. For the two-feature model comprising Energy and $\Delta \phi$ (Fig. 8a), $\Delta \phi$ exhibited a consistently higher mean SHAP value ($\approx$ 0.0190) relative to Energy ($\approx$ 0.0155). All five folds showed the same ordering, indicating a robust and stable contribution pattern across cross-validation splits. In the three-feature configuration XYZ + Energy + $\Delta \phi$ (Fig. 8b), $\Delta \phi$ again demonstrated the largest mean contribution ($\approx$ 0.0075), followed by Energy ($\approx$ 0.0050) and XYZ ($\approx$ 0.0045). The fold-wise trajectories maintained comparable spacing between features, underscoring the reproducibility of the relative importance hierarchy. For the two-feature model XYZ + $\Delta \phi$ (Fig. 8c), the spatial coordinates (XYZ) showed a marginally higher mean importance ($\approx$ 0.0060) than $\Delta \phi$ ($\approx$ 0.0055). The monotonic decrease observed across all folds from XYZ to $\Delta \phi$ further supports a consistent ranking of contributions within this feature set.

## Discussion

Only in recent years has quantum entanglement been explored as a potential source of additional information for event classification in PET imaging. Entangled annihilation $\gamma$-rays exhibit Compton scattering kinematics that differ from those of random $\gamma$-ray pairs of the same energy. While this behavior has been postulated and partially demonstrated as a tool for identifying true coincidences, the principle can also extend to dual-emission isotopes. In our cross-strip CZT system, event ordering ambiguities arise when multiple electrodes are activated by a single photon. We have previously addressed this challenge by developing methods to order electrode activation events using the relationship between position-derived and energy-derived polar scattering angles, employing both a rules-based approach^17^ and a ML approach^51^. These methods require MIPEs so that scattering vectors can be reconstructed and polar angles derived. Notably, this same requirement applies when investigating the effects of entanglement. While polar scattering angles themselves are unaffected by entanglement, the azimuthal scattering difference between entangled photons is. This provides an additional entanglement-sensitive feature for classification.

Event ordering ambiguities occur when multiple interactions (and thus multiple electrode activations) take place within a single crystal, as the available timing resolution (on the order of tens of nanoseconds) is insufficient to resolve their sequence. Correctly identifying the first interaction is critical for image quality, since this point defines one end of the LOR used in image reconstruction. By leveraging coincidence information, virtual LORs can be constructed for each possible ordering and tested against known Compton scattering kinematics. Ordering errors manifest as inconsistencies between measured energies and computed scattering angles. Likewise, random coincidences inherently produce such inconsistencies, as their assumed LOR – and, in the case of high-energy prompt $\gamma$’s, their assumed energy – is not a valid basis for scattering calculations. Thus, event ordering is inherently a dual problem: the same mechanisms that resolve the correct sequence of true coincidence events can also be used to reject randoms. In this work, our ML framework evaluates all possible configurations and identifies, when present, the most physically consistent true event.

Quantitatively, the incorporation of entanglement-sensitive features demonstrates measurable performance gains. The best-performing feature combination (E+$\Delta \phi$) achieved ROC-AUC values ranging from 0.87 to 0.95 across configuration classes, representing approximately 2 percentage points improvement over energy alone (ROC-AUC 0.85–0.95) for some classes. For random coincidence rejection (class 4), the E+$\Delta \phi$ combination achieved ROC-AUC of 0.95, demonstrating strong discriminative power for identifying invalid events. These metrics indicate that the azimuthal scattering angle difference provides complementary information beyond energy deposits alone, enhancing both event ordering accuracy and false coincidence rejection. While the ablation study (Table 2) demonstrated that E+$\Delta \phi$ achieved superior classification performance (ROC-AUC 0.87-0.95), SHAP importance analysis revealed that within this optimal combination (Fig. 8 (a)), $\Delta \phi$ contributed marginally higher discriminative weight ($\approx$0.019) than energy ($\approx$0.016), suggesting that quantum entanglement-encoded angular correlations provide critical disambiguation in cases where energy information alone is ambiguous. In our previous work, we achieved a test accuracy of 0.85 when distinguishing coincidences absorbed on one side and scattered into an absorption on the other (1P vs. 1C1P), a slightly simpler case^51^.

At first glance, it may appear surprising that energy alone (even accounting for the superior energy resolution of CZT) achieves such strong discrimination (0.85–0.95). However, this can be explained by considering the event selection: with a 50 keV window around 511 keV, the maximum energy that can be deposited by a Compton scatter is 340.7 keV. Thus, any event above this threshold must correspond to an absorption and must occur second. Moreover, $\gamma$-rays of this energy overwhelmingly favor shallow scatters (small polar scattering angles) with correspondingly small energy deposits – events that must necessarily occur first in the sequence. In cases of ambiguity, $\Delta \phi$ provides the most distinct information, as it is derived from positional data and is independent of energy, unlike polar scattering angles. The marginal contributions of spatial features (*xyz*) to any fold are consistent with the underparameterization hypothesis. A deeper network architecture might better leverage spatial information, but for the current model, using derived quantities from these parameters is more effective and computationally efficient. The incremental 2 percentage point improvement achieved by adding $\Delta \phi$ to energy underscores that while energy provides the primary discriminative signal, entanglement-encoded angular correlations offer a meaningful secondary mechanism for enhancing classification accuracy, particularly for borderline cases where energy information alone may be ambiguous.

While entangled annihilation photon populations exhibit a specific $\Delta \phi$ distribution (greater relative frequency about $\Delta \phi =\pm 90^\circ$), measurements of individual $\Delta \phi$s cannot be used to deterministically classify entanglement status. This limitation is because non-entangled photon pairs (such as randoms) can scatter with arbitrary $\Delta \phi$ including within the $\pm 90^\circ$ region. It is only the case that $\pm 90^\circ$ scatters are more likely for entangled than non-entangled. And yet, we observe relatively strong performance for the random detection event (class 4 label) and weak performance for discriminating between hit configurations for true events (class labels 0-3) for the $\Delta \phi$-only model. This phenomenon is likely due to partial geometric symmetry of $\Delta \phi$ across configurations. For a given $C\rightarrow P$ event, swapping the ordering of C and P drastically changes the observed $\theta$ but only flips the sign of $\phi$. For a true DCSc where $\Delta \phi$ holds physical meaning, the four configurations are in two pairs with identical $\Delta \phi$ within each pair. For a random DCSc where $\Delta \phi$ holds no physical meaning, the $\Delta \phi$ response and $\theta$ responses mix and contaminate each other, breaking this symmetry. So, based solely on $\Delta \phi$, we postulate such symmetries allow for good classification of true (configuration independent) vs random events with poor classification of which configuration was true.

Working with dual-emission isotopes, such as Sc-44, poses unique challenges due to the need to disentangle the prompt $\gamma$-ray signal from the annihilation $\gamma$-ray signal. Prompt $\gamma$-rays with energies greater than 511 keV can contaminate the signal space by scattering into this energy range—thereby mimicking annihilation $\gamma$-rays—or by depositing confounding amounts of energy across one or more detector interactions. Imaging dual-emission isotopes can be more difficult than working with mixtures of isotopes, where each isotope has a distinct emission profile and where events can often be separated using timing resolution. In dual emitters, however, prompt and annihilation emissions can occur within sub-timing-resolution differences, preventing effective separation by conventional coincidence timing techniques. Consequently, alternative mechanisms must be leveraged for discrimination. Beyond energy information and the correlations encoded in Compton scattering angles, no other physical mechanism exists for classification on an event-by-event basis – aside from quantum entanglement. In this work, we demonstrate that incorporating this additional layer of physics enhances classification performance, by upwards of 2 percentage points for some classes ((E) vs (E+$\Delta \phi$) folds).

Our previous work with entanglement-informed classification strategies faced the challenge of poor statistics when selecting only the most promising candidates – those with polar scattering angles near $\theta = 81.7^{\circ }$. In this study, we do not impose this filtering requirement, thereby substantially increasing the usability of entanglement-derived information. Nevertheless, techniques that rely on entanglement correlations (as currently understood) require that scattering information be available so that differences in azimuthal angle can be computed. This intrinsically limits the set of coincidences to which such methods can be applied. While we have shown that 1C1P events are the most common interaction type for the CZT PET scanner used in this study^17,52^, this distribution may differ in other system designs or detector materials. Furthermore, the performance of the ML framework – and the discriminative power of the features – depends strongly on system-specific factors such as energy resolution and spatial resolution. In addition, our dataset generation relied on several simplifying assumptions which, although valid, may introduce subtle biases into the training set. Finally, the present work was limited to Sc-44; other dual-emission isotopes with different prompt $\gamma$-ray energies may exhibit distinct behaviors under the same approach, necessitating further investigation.

Although the proposed approach demonstrates promising performance, several limitations should be acknowledged. First, the experiments were conducted on a dataset of limited size and scope, which may restrict the generalizability of the findings to broader clinical or real-world settings. Second, the evaluation relied primarily on F1-score and ROC-AUC, which, while informative, may not fully capture other important aspects such as robustness to noise, interpretability, or clinical usability. Third, although the model was implemented and trained on a high-performance workstation, the exploration of alternative architectures, hyperparameter settings, or more extensive ablation studies was constrained by computational resources. Finally, external validation on independent datasets and testing in real-world clinical workflows were not performed, which limits conclusions about domain transferability and practical deployment.

An important next step will be validating the ML model with real experimental datasets from the CZT system. While simulation provides ground truth information and controlled conditions, it cannot fully capture system artifacts such as crosstalk or noise. Using real measurements in the training and evaluation pipeline will enable robustness and establish the practical feasibility of entanglement-informed classification strategies. Similarly, incorporating training sets comprised of multiple studies of different dual-emission isotopes (with prompt $\gamma$-rays at various energy levels) is necessary for generalizability across CZT PET systems and radionuclide classes. Finally, exploring additional ML architectures such as graph neural networks (GNNs) or support vector machine (SVM), may capture better event topology. As GNNs use nodes and graphs, they may offer a natural representation of MIPEs; on the other hand, SVMs may provide improved discrimination in lower-dimensional feature spaces.

## Conclusion

In this work, we present a ML–based framework for event sequencing and configuration selection in a dual-panel CZT PET system. The approach leverages a structured representation of MIPEs and a permutation-invariant architecture that jointly evaluates competing configurations, selecting the one most consistent with the underlying physics. By including a null class for unfavorable candidates, the method simultaneously acts as a robust rejection of invalid events, which is essential for reliable event recovery in practical deployments. A systematic feature ablation with five-fold cross-validation showed that energy + $\Delta \phi$ correlations consistently achieved the highest performance among tested feature sets in this study, with ROC–AUC values ranging from approximately 0.87–0.95 across classes 0-4. Specifically, the inclusion of $\Delta \phi$ with energy improved discrimination performance by approximately 2 percentage points across some configuration classes compared to energy alone (ROC-AUC 0.85–0.95), demonstrating that incorporating entanglement-sensitive angular features provides measurable enhancement in both event ordering accuracy and random coincidence rejection. Notably, the random event class (class 4) achieved particularly strong discrimination with ROC-AUC of 0.95 using E+$\Delta \phi$, underscoring the specific value of quantum-encoded angular correlations for identifying invalid coincidences.Energy alone also provided strong performance, while the addition of spatial coordinates offered complementary benefits for interpretability and potential robustness to geometric variations. These findings suggest that angular separation and energy are particularly informative descriptors for event classification in this setting. This highlights a practical trade-off: while (E+$\Delta \phi$) maximizes raw discrimination, integrating spatial context can enhance stability. The ML approach yields reliable performance at both the row level (candidate configuration classification) and the sample level (overall event decision). Future validation on experimental datasets and across different isotopes and system designs will be essential to assess generalizability and practical feasibility.

## Data Availability

The dataset generated and/or analysed during the current study are available in the “ml-pet-event-classification-data” repository. Link: https://github.com/RIL-Hub/ml-pet-event-classification-data
